# Robotic microsurgery

**DOI:** 10.1186/1753-6561-9-S3-A94

**Published:** 2015-05-19

**Authors:** Philippe Liverneaux

**Affiliations:** 1 Department of Hand Surgery, Strasbourg University Hospitals, F-67493, Illkirch, France

## 

Conventional microsurgery requires large incisions and extensive dissection while it is performed in a small operating field. In this context, the concept of endoscopic microsurgery appears to be a logical evolution.

We rely on four years of practical experience using the Da Vinci robot at the European Institute of telesurgery in Strasbourg, France. To date, 50 patients have been operated on by our Hand Surgery and Peripheral Nerve Department. Endoscopic Microsurgery combines the properties of microsurgery, endoscopic surgery and telesurgery. Not only does it permit to magnify the vision of the operating field, but also to multiply the operator’s hand movements, and all this by minimally invasive approaches. Its evolution necessitates the development of a dedicated robot and specific instrumentation capable of handling such procedures.

